# Functional Analysis of PxylPBP2 Responding to Repellent Activity of Natural Pyrazine Against Diamondback Moth

**DOI:** 10.3390/insects17070708

**Published:** 2026-07-08

**Authors:** Yan-Lu Zhou, Zi-Xian Wang, Xian-Wen Wang, Bu-Guo Wang, Qing Li, Lan Wang, Min Liao, Hai-Qun Cao, Quan Gao

**Affiliations:** 1 Anhui Province Engineering Laboratory for Green Pesticide Development and Application, School of Plant Protection, Anhui Agricultural University, Hefei 230036, China; 18214895676@163.com (Y.-L.Z.); 18225523762@163.com (Z.-X.W.); 19577305475@163.com (X.-W.W.); wbg986798196@126.com (B.-G.W.); 15753931981@163.com (Q.L.); 19397278328@163.com (L.W.); liaomin3119@126.com (M.L.); caohq@ahau.edu.cn (H.-Q.C.); 2 Key Laboratory of Agri-Products Quality and Biosafety (Anhui Agricultural University), Ministry of Education, Hefei 230036, China

**Keywords:** *Plutella xylostella*, PxylPBP2, 2,3-dimethyl-6-(1-hydroxy)-pyrazine, repellent activity, binding mechanism

## Abstract

2,3-dimethyl-6-(1-hydroxy)-pyrazine showed significant repellent activity to diamondback moth (*Plutella xylostella*), the PxylPBP2 was considered as the potential molecular target, though this has not been validated. In this study, the repellent rate was significantly decreased by about 20% when the *PxylPBP2* was silenced using RNAi. There was obvious interaction between 2,3-dimethyl-6-(1-hydroxy)-pyrazine and PxylPBP2, with a Kd (Dissociation constant) value of 7.78 μmol/L via microscale thermophoresis, while the value was changed to 4.45 μmol/L when the PxylOR31 (Pxyl, *Plutella xylostella*; OR31, Odorant receptor 31) was introduced to this compound and ligand system. Moreover, the RMSD (Root mean square deviation) value of pyrazine + PxylPBP2 + PxylOR31 treatment ranged between 0.030 and 0.125 nm; the average number of hydrogen bonds and Van der Waals interaction value can be used to summarize that there was a stronger interaction between pyrazine and PxylPBP2. Furthermore, the ILE122 (L-Isoleucine at 122 site) was an important target site during the binding process between PxylBPB2 and ligands, which was validated by performing a fluorescence competitive binding experiment. Finally, the PxylPBP2 showed a strong ability to bind to *α*-pinene and *β*-ionone with the Ki values of 10.54 μmol/L and 11.74 μmol/L, respectively. These data provide a creative strategy for developing novel insecticides, which is also important for achieving the green control of *P. xylostella* and delaying the development of resistance.

## Simple Summary

The extensive use of chemical insecticides has led to the development of serious resistance by the diamondback moth, but developing a novel active ingredient is an effective strategy to solve this problem. Based on preliminary laboratory experiments, we identified 2,3-dimethyl-6-(1-hydroxy)-pyrazine as a substance with significant potential to act as a repellent for the diamondback moth, and PxylPBP2 (Pxyl, *Plutella xylostella*; PBP2, Pheromone binding protein 2) was considered as a potential target. Hence, we validated the role of *PxylPBP2* in the moth’s response to the repellent activity via RNAi, and the binding mechanism and characteristics for interaction between PxylPBP2 and pyrazine were assayed using a microscale thermophoresis test and molecular dynamics simulation. In addition, the binding ability was determined using a mutant protein and a fluorescence competitive binding assay. These data provide an interesting strategy for achieving the green control of *P. xylostella* and delaying the development of resistance in diamondback moths.

## 1. Introduction

The diamondback moth (DBM, *Plutella xylostella*) is an important Lepidoptera pest that mainly damages the cruciferous crops during its larval period, causing serious losses [1]. There distribution characteristics of this species have clear regional and temporal variability; three ranges were validated, namely the stable overwintering zone (the South China and Central China regions), the source of overwintering pests (a local area in North China), and the no-overwintering zone (the DBM in the main regions of Northeast and North China); Henan Province was determined to be a boundary region [2]. The DBMs source present in North China during Spring mainly originates from migratory populations from South China, as DBMs do not overwinter in this region; this migration causes wider-ranging and longer-lasting damage to crops [3]. Currently, the utilization of chemical insecticides is the main control method for DBMs, while insecticide resistance has become the most important problem to overcome. The resistance of DBMs to some common chemical ingredients, such as abamectin, lambda-cyhalothrin, indoxacarb, chlorfenapyr, and chlorantraniliprole, has quickly increased [4,5,6]. A field-collected population of *P. xylostella* from Aichi prefecture in Japan was found to exhibit strong (>150-fold) resistance to emamectin benzoate when compared with a laboratory susceptible strain [7]. Moreover, resistance ratios of DBMs collected from cabbage and cauliflower fields at Bapatla, Chirala, Ponnur, and Tenali (Andhra Pradesh, India) ranged from 9.8 to 13.1 for tolfenpyrad and 5.3 to 10.2 for emamectin benzoate; biochemical assays demonstrated that tolfenpyrad resistance was primarily associated with increased glutathione-*S*-transferase activity, while emamectin benzoate resistance was largely mediated through carboxylesterase activity [8]. Serious resistance resulted from the excessive application of imidacloprid and chlorantraniliprole, which significantly decreases the control effect [9]. Field populations of *P. xylostella* developed high resistance to chlorantraniliprole within 3 years of the pesticide’s introduction [10], with the number in the Arthropod Pesticide Resistance Database, which tracks insecticide resistance, being greater for *P. xylostella* than for any other arthropod (Arthropod Pesticide Resistance Database, Michigan State University). Overall, the DBM has developed resistance to at least 97 active ingredients worldwide; these patterns underscore the rapid evolution of the pest’s resistance under intensive selection pressure in diverse agroecosystems [11] (APRD, Arthropod Pesticide Resistance Database, *Plutella xylostella* resistance records, Michigan State University).

Recently, the use of botanical insecticides has been considered as an alternative strategy to reduce the utilization of chemical insecticides and delay the development of resistance, showing unique mechanisms of action and application advantages. The essential oil of *Cymbopogon schoenanthus* and neem oil showed significant antifeedant activity, inhibiting the adult eclosion effect of *P. xylostella*, which can expand the application range due to the synergistic effect [12]. Among the more than 95 plant species whose extracts have been studied for insecticidal activity, *Azadirachta indica*, *Capsicum annuum*, *Nicotiana tabacum*, and *Tagetes erecta* have been the most researched [13]. The aqueous extract of *Ruta graveolens* has an obvious insecticidal effect and the potential to act as a repellent for *P. xylostella*, with trans-anethole identified as the main active component [14,15], though the essential oil also promotes high growth inhibition in third-instar larvae [16]. Aqueous extracts of the leaves of *Alibertia intermedia* and *A. sessilis* negatively affected the development of *P. xylostella* in all phases of its life cycle, prolonging the larval period and causing larval mortality of 43.59% and 50.2%, respectively [17]. Some aqueous botanical extracts obtained via maceration with dry leaves of *Schinus terebinthifolius*, *Ludwigia tomentosa*, *L. longifolia*, *L. sericea*, and *L. nervosa* demonstrated insecticidal potential against newly emerged larvae of *P. xylostella*, with mortality ranging from 14.00% to 38.00% [18]. Topical application to adult female parasitoids versus second instars of *P. xylostella* showed that thymol was about 62 times less toxic to the parasitoid than to the host larvae with 24 h of treatment at LD_50_ (Median lethal dose) of 52 and 8 mmol/L, respectively [19]. Thus, ethyl acetate extract of *Consolida ajacis* seed had obvious contact toxicity with an LC_50_ (Median lethal concentration) value of 5.05 mg/mL, and the main active compound was identified as ethyl linoleate [20]. Additionally, utilization of RNAi-based biopesticides has been developed as a novel strategy to control agricultural pests, which has some key advantages, including high efficacy, minimal off-target effects, and compatibility with integrated pest management systems [21]. It has been reported that when Hemolin *dsRNA* was injected into *Hyalophora cecropia* pupae, the transgenerational embryonic abnormalities and mortality were induced [22]. RNAiSSANCE Ag is progressing toward commercialization of another foliar-applied RNAi-based pesticide targeting *P. xylostella* [23].

Insects use olfactory sensilli to receive and recognize pheromones and other odors, including the components with repellent activity, with these factors playing a key role during foraging and mating [24]. Antennae are the main organ used in this process, containing a large number of sensilla specialized from epidermal cells, with odorant-binding proteins (OBPs) and odorant receptors (ORs) being the most important molecular targets for insecticides [25,26]. OBPs can be linked with the odor in combination after the compound has been transferred into the lymph; then, the OR can be activated to recognize the combination or odor to guide related insect behavior, changing the chemical signal into an electrical signal to be conducted to the nervous center [27,28]. OBPs can be classified as long chains (about 160 amino acids), moderate chains (about 120 amino acids), or short chains (about 100 amino acids) based on the number of amino acid residues in the first structure, while they can also be classified as classic OBP (6 conserved cysteine residues), plus-C OBP (more than six cysteine residues), or minus-C OBP (four cysteine residues) according to the difference in the cysteine residue number [29,30]. A total of 24 genes that can encode OBPs were identified via the sequencing of *P. xylostella* antennae, including three pheromone-binding proteins (PBPs) and three general OBPs (GOBPs) [31,32]. Significant tissue-specific expression patterns of the *P. xylostella* OBP gene family were identified: *PxylOBP3* was only expressed in the antennae of adults, *PxylOBP9* was found in the antennae and reproductive organs, and *PxylOBP19* was widely expressed in all tissues, being defined as a plus-C OBP containing nine cysteines [33]. While 39 candidate OBPs were identified from genome and transcriptome sequence data, the expression levels of several PxylOBPs regulated feeding and mating behavior, providing new insights that clarified the mechanism of OBP regulation in insect chemical communication procedures [34]. As farnesol showed obvious attractant activity toward *P. xylostella* larvae, RNAi technology was used to indicate the critical role of PxylGOBP2 in recognizing farnesol, which also revealed the importance of residues such as Thr9 (Threonine 9), Trp37 (Tryptophan 37), and Phe118 (Phenylalanine 118) in PxylGOBP2 binding to farnesol [35].

Our previous study showed that the natural product 2,3-dimethyl-6-(1-hydroxy)-pyrazine has obvious repellent activity to *P. xylostella* adults, and it can also affect OBPs and ORs in the antennae [36,37]. A total of 25 OBPs were obtained from the transcriptomic sequence data via differentially expressed odor perception-related gene analysis, and PxylPBP2 may be the most important one in terms of the potential repellent activity of pyrazine [38]. In this study, the PxylPBP2 relationship with the potential repellent activity of 2,3-dimethyl-6-(1-hydroxy)-pyrazine was validated via RNAi. Moreover, the binding mechanism between PxylPBP2 and pyrazine was assayed using a microscale thermophoresis test and molecular dynamics simulation. Subsequently, the mutant protein PxylPBP2^I122A^ was obtained, and a fluorescence competitive binding assay was used to validate the binding ability between the target and the compound. Finally, ten common volatile compounds were selected to measure the recognition characteristics of PxylPBP2. Our findings demonstrate that PxylPBP2 may be important to trigger repellent activity of 2,3-dimethyl-6-(1-hydroxy)-pyrazine, which was also identified as a candidate target of novel green pesticide ingredients, and provide a creative strategy for both achieving the comprehensive control of *P. xylostella* and delaying the development of resistance.

## 2. Materials and Methods

### 2.1. Insect Rearing

The *P. xylostella* colony was established and preserved at the School of Plant Protection, Anhui Agricultural University, China. Larvae and adults were, respectively, fed with standard Chinese cabbage and 10% (*v*/*v*) honey solution, and continuously maintained in an environment-controlled growth incubator at 27 °C with a 16 h:8 h (light/darkness) photoperiod and 80% relative humidity. Pupae were manually collected and enclosed in plastic cages with gauze modified in our laboratory (15 cm × 15 cm × 25 cm). *P. xylostella* adults were used for the experiments conducted in this study.

### 2.2. Generation of PxylPBP2 Interference Mutant

#### 2.2.1. Design for the Primers and PCR Amplification of RNAi

The dsRNA (Double-stranded RNA) primers were designed and synthesized based on the sequence of *PxylPBP2* using Primer Premier 5.0 software (Premier Biosoft International, Palo Alto, CA, USA); use of fluorescent quantitative primers should be avoided during this procedure. Then, the T7 RNA polymerase promoter sequence was added to the 5′ end of the primers, as shown in Table S1, together with the green fluorescent protein (GFP) gene as a negative control.

Subsequently, PCR results were determined using 1% agarose gel electrophoresis after the amplification process had been accomplished via the standard procedure and the reaction system (Table S2). Then, the correct target fragments were re-collected and transferred into a *DH5α* cell, and the positive colony was used to extract the plasmid after the suspension was sequenced as correct. Finally, the recollected product was saved at −20 °C via the standard method, and it was used as the template for *dsRNA* synthesis.

#### 2.2.2. Synthesis for dsRNA and Product Recollection

The synthesis was assayed using the above-mentioned product as a template using the related kit referred to in the T7 RiboMAXTM Express Large Scale RNA Production System instructions (Promega, Southampton, UK); the reaction system is shown in Table S3. Firstly, the system was slightly mixed and cultivated at 37 °C for 30 min, and 1 μL of RQ1 RNase-Free DNase (Promega, Southampton, UK) was added and continuously cultivated at 37 °C for 15 min. Then, 21 μL of a mixture of phenol (pH 4–5), chloroform, and isopentanol with a volume ratio of 125:24:1 was used to extract from the suspension. The supernatant was transferred to another EP tube with the same volume of chloroform, and isopentanol (24:1) was extracted after the oscillatory and centrifugal processes, which were performed once. Next, a 10% volume ratio of sodium acetate (3 mol/L, pH 5.2) and isopropanol was added, and the mixture was centrifuged for 10 min after being placed on ice for 5 min. Lastly, the supernatant was extracted and mixed with 1 mL 70% ethanol solution to wash the precipitate; then, 20 μL of nuclease-free water was used to dissolve the *dsRNA*, which was saved at −80 °C.

#### 2.2.3. Silencing by RNAi and Repellent Activity Assay

In this study, *P. xylostella* (randomly 25 males and 25 females) first instar pupae with similar body color and individual size were used to assay the RNAi test. The *dsRNA* (300 ng) was injected into the site between the third and fourth segments of the pupa abdomen using the Nanoliter 2010 microinjection system (WPI, Sarasota, FL, USA). A negative control treatment was injected with 300 ng of *dsGFP*, which was performed in triplicate for each treatment. All of the pupae were collected and cultivated via the above-mentioned method. After 72 h, the *P. xylostella* (5 males and 5 females, all randomly selected) were selected to detect the silencing efficiency via RT-qPCR.

Then, the glass Y-tube olfactometer (Designed by our team; tube length, 20 cm; inner diameter, 1 cm; an angle between the two tubes, 75°; straight pipe, 20 cm) was used to determine the repellent activity of 2,3-dimethyl-6-(1-hydroxy)-pyrazine (obtained from our laboratory) against *P. xylostella* adults [37]. A total of 30 adults (15 males and 15 females, all randomly selected) were used in each replicate, and every treatment was replicated three times.

### 2.3. Binding Mechanism Between PxylPBP2 and 2,3-Dimethyl-6-(1-hydroxy)-pyrazine

#### 2.3.1. Microscale Thermophoresis Analysis to 2,3-Dimethyl-6-(1-hydroxy)-pyrazine and PxylPBP2

Microscale thermophoresis (MST) is used to study various intermolecular interactions and processes, such as the formation of ligand-target complexes, aggregation, and acid dissociation, as well as to track reactions and perform kinetic and thermodynamic analysis, which has become increasingly popular and competitive with alternative techniques [39,40]. In this study, binding reactions of 2,3-dimethyl-6-(1-hydroxy)-pyrazine with PxylPBP2 and PxylOR31 recombinant proteins were measured via the MST test in a Monolith NT.115 instrument (NanoTemper technologies, München, Germany). To construct His-fused plasmids, PxylPBP2 or PxylOR31 was inserted into the pET28a(+) vectors, then converted to pET28a(+):PxylPBP2 or PxylOR31 in *E. coli* BL21(DE3). The recombinant protein PxylPBP2 or PxylOR31-His was purified with Ni-NTA agarose from *E. coli* BL21(DE3). The PxylPBP2 or PxylOR31-His buffer was displaced via 1× PBS buffer and fluorescently labeled. A range of concentrations of 2,3-dimethyl-6-(1-hydroxy)-pyrazine in the assay buffer were incubated with labeled PxylPBP2 or PxylOR31-His (10 µM) (1:1, *v*/*v*) for 15 min. The samples were loaded into the NT Label-Free standard capillaries and measured with 100% light-emitting diode power and 40% MST power. The Kd-fit (Dissociation constant) function of the Nano Temper analysis software (MO. Affinity Analysis v.1.5.41) was used to fit the curve and calculate the value of the Kd. The experiments were repeated three times.

#### 2.3.2. Molecular Dynamics Simulation

In order to clarify the structural stability and interaction characteristics between PxylPBP2 or PxylOR31 proteins and the ligand of 2,3-dimethyl-6-(1-hydroxy)-pyrazine, molecular dynamics simulation was used to evaluate the RMSD (Root mean square deviation), number of hydrogen bonds, and van der Waals force and electrostatic interactions of different systems. The software GROMACS 2020.6 was used to assay a 100 ns simulation on the system of PxylPBP2 + 2,3-dimethyl-6-(1-hydroxy)-pyrazine, PxylOR31 + 2,3-dimethyl-6-(1-hydroxy)-pyrazine, 2,3-dimethyl-6-(1-hydroxy)-pyrazine + PxylPBP2 + PxylOR31, with the CHARMM36 force field. The model was TIP3P with a step length of 2 fs, a temperature of 300 K, and a pressure of 1 bar.

### 2.4. Construction of the Mutant Protein PxylPBP2^I122A^ and the Fluorescence Competitive Binding Assays

#### 2.4.1. Selection of the Mutant Site

The sequence of PxylPBP2 was inserted into SnapGene (GSL Biotech LLC, San Diego, CA, USA), and the amino acids of seven potential key binding sites were replaced by alanine based on the results of molecular docking. Then, the new sequences were inserted into Swiss-Model (swissmodel.expasy.org) to perform modeling and prediction, and the PDB files of seven protein structures were downloaded to perform in-depth analysis after the binding energy was calculated using molecular docking software.

#### 2.4.2. Cloning and Vector Construction for the Mutant Protein PxylPBP2^I122A^

The forward primer was AACTGGTGACCATCGCCCACGAGTGCGAGCAGGGC, and the reverse primer was GGCGATGGTCACCAGTTGTTTCGCCATCGTGT, respectively, and they were designed using CE (Clone Express Design1.04) software (Vazyme, Nanjing, China). Subsequently, PCR amplification was performed using the Mut Express^®^ II Fast Mutagenesis Kit V2 (Vazyme, Nanjing, China), and the reaction system is shown in Table S4. The reaction procedure was determined to be an initial denaturation step at 95 °C for 30 s, 30 cycles of denaturation at 95 °C for 15 s, annealing at 70 °C for 15 s, and extension at 72 °C for 3 min, followed by 72 °C for 5 min, and the sample was finally stored at 4 °C. Then, the product was validated via 1% agarose gel electrophoresis, and the correct plasmids were used to assay the next Dpn I digestion procedure using the reaction system at 37 °C for 2 h (Table S5).

Subsequently, after detection and validation, the product was placed into the LB (Luria–Bertani) medium containing Amp^+^ (Ampicillin), which was cultivated at 37 °C and 200 rpm (Revolutions per minute); then, the solution was sequenced according to the standard method. Finally, the extraction of the candidate target fragment plasmid was performed using the FastPure^®^ plasmid mini kit (Vazyme, Nanjing, China) with a related protocol; the obtained DNA solution was stored at −20 °C after the concentration had been determined. The recombination reaction was performed after the optimal amount of Dpn I digestion product was selected (determined according to the number of fragment base pairs), and the reaction system was prepared on ice (Table S6). The system was gently mixed well without shaking, and the reaction solution was collected at the bottom of the tube after brief centrifugation. Then, the reaction was completed at 37 °C for 30 min, and the product was immediately cooled on ice and finally stored at −20 °C. Finally, the recombination product was identified, and the plasmid was extracted via the standard method.

#### 2.4.3. Fluorescence Competitive Binding Experiment for Mutant Protein PxylPBP2

Firstly, the PxylPBP2^I122A^ protein was purified and dialysis-refolded via the method established in this study, and a concentration was determined that satisfied the next test. Then, the fluorescence competitive binding experiment was assayed to validate the binding ability between 2,3-dimethyl-6-(1-hydroxy)-pyrazine and PxylPBP2^I122A^, which was also compared with PxylPBP2.

### 2.5. Recognition Characteristics of PxylPBP2 with Some Volatile Compounds

To gain an in-depth understanding of the function of PxylPBP2, ten common volatile compounds were selected to measure the recognition characteristics. Volatile compounds used in this test included 1-hexanol, linalool, benzyl alcohol, benzaldehyde, *α*-pinene, ocimene, myrcene, *β*-ionone, *β*-citronellol, and flavone, and related data are shown in Table S7. The test was completed using the above-mentioned fluorescence competitive binding experiment method.

## 3. Results

### 3.1. PxylPBP2 Is Involved in the Repellent Activity of 2,3-Dimethyl-6-(1-hydroxy)-pyrazine

To validate the response of PxylPBP2 to 2,3-dimethyl-6-(1-hydroxy)-pyrazine, the RNAi procedure was assayed. Firstly, the silencing efficiency was calculated via comparison with the treatment of dsGFP; the expression level was significantly decreased (*p* < 0.01), and the value was 45.65% (Figure S1). We determined that the *PxylPBP2* gene was successfully disrupted, which was confirmed in the next experiment.

**Figure 1:**
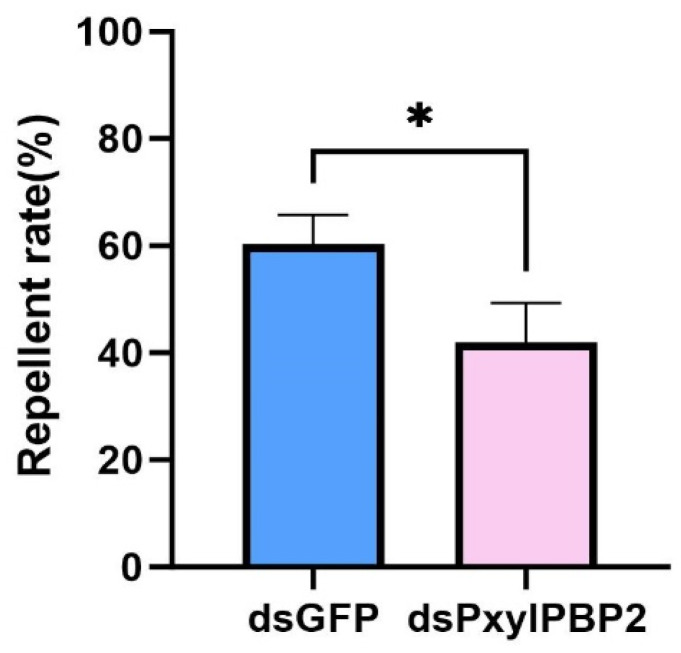
The repellent activity of 2,3-dimethyl-6-(1-hydroxy)-pyrazine for adult *P. xylostella* after RNAi. Differences between groups are denoted by stars, with *p* < 0.05 representing the threshold for statistical significance.

When the *P. xylostella* reached the adult stage, the glass Y-tube olfactometer was used to assay the repellent activity of 2,3-dimethyl-6-(1-hydroxy)-pyrazine. As shown in Figure 1, the repellent rate for *P. xylostella* adults injected with *dsPxylPBP2* was 41.88%, which was identified as level III. While the rate for *dsGFP* treatment was 60.31% at level IV, there was a significant difference between these treatments (*p* < 0.05). The repellent activity was notably decreased due to the silencing of *PxylPBP2*, suggesting that PxylPBP2 plays an important role in the process through which *P. xylostella* recognizes pyrazine, as well as participating in olfactory perception or behavioral regulation.

### 3.2. MST Characteristics Shared 2,3-Dimethyl-6-(1-hydroxy)-pyrazine and the Olfactory System Protein

1104YS-1-His (PxylOR31) and 104YS-2-His (PxylPBP2) were successfully expressed and purified (Figure S2), and the concentrations were determined to be 0.97 mg/mL and 0.62 mg/mL, respectively, satisfying the MST experiment. Subsequently, these two proteins were labeled with small fluorescent molecules using the MonolithTM RED-NHS kit, which was placed on ice and immediately assayed for the next stage of MST detection.

**Figure 2:**
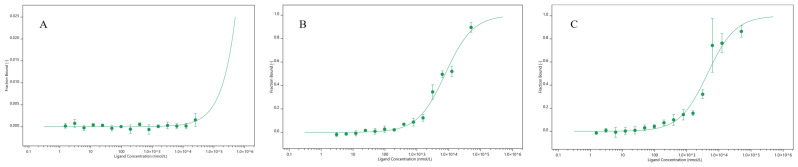
MST characteristics shared between the olfactory system protein and 2,3-dimethyl-6-(1-hydroxy)-pyrazine. (**A**) MST curve between PxylOR31 and 2,3-dimethyl-6-(1-hydroxy)-pyrazine. (**B**) MST curve between PxylPBP2 and 2,3-dimethyl-6-(1-hydroxy)-pyrazine. (**C**) MST curve between PxylOR31, PxylPBP2 and 2,3-dimethyl-6-(1-hydroxy)-pyrazine.

As shown in Figure 2A, the fraction bound value between the PxylOR31 protein and the pyrazine ligand changed until the concentration of the pyrazine ligand was 10^−4^ mol/L. Hence, there was no obvious interaction between the PxylOR31 protein and the pyrazine ligand. In turn, the fraction bound value between 2,3-dimethyl-6-(1-hydroxy)-pyrazine and PxylPBP2 changed from 0 to 1.0 with an increase in the concentration of the pyrazine ligand; when the concentration of pyrazine was 10^−4^ mol/L, the change was significant (Figure 2B). The Kd value was calculated as 7.78 ± 1.46 μmol/L, so interaction between 2,3-dimethyl-6-(1-hydroxy)-pyrazine and PxylPBP2 was confirmed.

Moreover, the change trend between 2,3-dimethyl-6-(1-hydroxy)-pyrazine and PxylOR31, PxylPBP2 was similar to the data between PxylPBP2 and pyrazine, with a Kd value of 4.45 ± 1.12 μmol/L (Figure 2C). The interaction between PxylOR31, PxylPBP2, and 2,3-dimethyl-6-(1-hydroxy)-pyrazine was significant, suggesting that pyrazine showed repellent activity by affecting the function of PxylOR31 and PxylPBP2 simultaneously.

### 3.3. Molecular Dynamics Simulation of the Interaction Between the Olfactory System Protein and 2,3-Dimethyl-6-(1-hydroxy)-pyrazine

**Figure 3:**
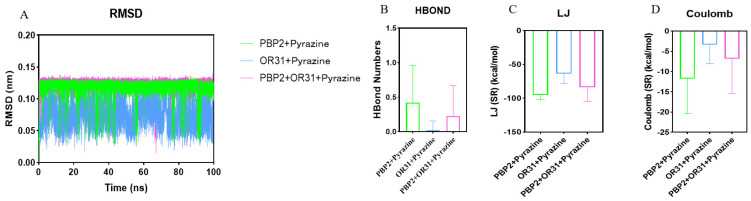
Molecular dynamics simulation of the interaction between the olfactory system protein and 2,3-dimethyl-6-(1-hydroxy)-pyrazine. (**A**) RMSD value between PxylOR31, PxylPBP2 and 2,3-dimethyl-6-(1-hydroxy)-pyrazine. (**B**) Hydrogen interaction between PxylOR31, PxylPBP2 and 2,3-dimethyl-6-(1-hydroxy)-pyrazine. (**C**) Van der Waals interaction between PxylOR31, PxylPBP2 and 2,3-dimethyl-6-(1-hydroxy)-pyrazine. (**D**) Electrostatic interaction between PxylOR31, PxylPBP2 and 2,3-dimethyl-6-(1-hydroxy)-pyrazine.

As shown in Figure 3A, the RMSD value of 2,3-dimethyl-6-(1-hydroxy)-pyrazine fluctuated around 0.125 nm, suggesting that the conformation of pyrazine was relatively stable due to the existence of PxylPBP2 with a limited fluctuation range. However, the RMSD value ranged between 0.030 and 0.125 nm when the PxylOR31 was present, suggesting that the flexibility of the pyrazine conformation was more significant and may correlate with the looseness of the binding site. Simultaneously, it was found that the average number of hydrogen bonds was 0.4 and 0.2 for the treatment of PxylPBP2 and 2,3-dimethyl-6-(1-hydroxy)-pyrazine and PxylOR31, PxylPBP2, and 2,3-dimethyl-6-(1-hydroxy)-pyrazine, respectively, while the value for the treatment of PxylOR31 and 2,3-dimethyl-6-(1-hydroxy)-pyrazine was nearly 0 for the 100 ns simulation (Figure 3B and Figure S3). The hydrogen interaction occurred between the PxylPBP2 and pyrazine, suggesting that the hydrophobic interaction was the main type of interaction.

Simultaneously, the average Van der Waals interaction was calculated as −95 kcal/mol between PxylPBP2 and 2,3-dimethyl-6-(1-hydroxy)-pyrazine, and the value between PxylOR31, PxylPBP2, and 2,3-dimethyl-6-(1-hydroxy)-pyrazine was −80 kcal/mol, with all values ranging from −80 to −100 kcal/mol (Figure 3C and Figure S4). In contrast, the average value of another treatment was −60 kcal/mol, with all values ranging from −25 to −75 kcal/mol. There was a strong Van der Waals interaction between 2,3-dimethyl-6-(1-hydroxy)-pyrazine and PxylPBP2, further clarifying that the hydrophobic environment differs between the PxylOR31 and PxylPBP2 binding pockets.

The electrostatic interaction between 2,3-dimethyl-6-(1-hydroxy)-pyrazine and PxylPBP2 ranged from 0 to −30 kcal/mol, and the average value was −12 kcal/mol; a similar range was obtained for the treatment of PxylOR31, PxylPBP2, and 2,3-dimethyl-6-(1-hydroxy)-pyrazine, with an average value of −7 kcal/mol (Figure 3D and Figure S5). Furthermore, the average value for the treatment of PxylOR31 and 2,3-dimethyl-6-(1-hydroxy)-pyrazine was −3 kcal/mol, with all values ranging from 0 to −10 kcal/mol.

Hence, there was a stronger interaction between PxylPBP2 and 2,3-dimethyl-6-(1-hydroxy)-pyrazine compared with the PxylOR31 receptor, while the binding energy was higher and the ligand conformation was more stable. PxylPBP2 was the main combination protein for pyrazine, having a stable binding pocket and strong non-covalent interaction.

### 3.4. Combination Mechanism Between PxylPBP2 and 2,3-Dimethyl-6-(1-hydroxy)-pyrazine

The binding energy changed from −22.61 to 5.62 kcal/mol between 2,3-dimethyl-6-(1-hydroxy)-pyrazine and PxylPBP2 when the isoleucine (ILE122) of No. 1222 was changed to alanine, while the key sites of PHE146 (Phenylalanine 146), PHE40, LEU80 (Leucine 80), TRP65 (Tryptophan 65), MET33 (Methionine 33), and LEU36 also simultaneously involved site-directed mutagenesis as a candidate target (Table S8). Hence, the ILE122 site plays an important role in the combination procedure between PxylPBP2 and ligands.

**Figure 4:**
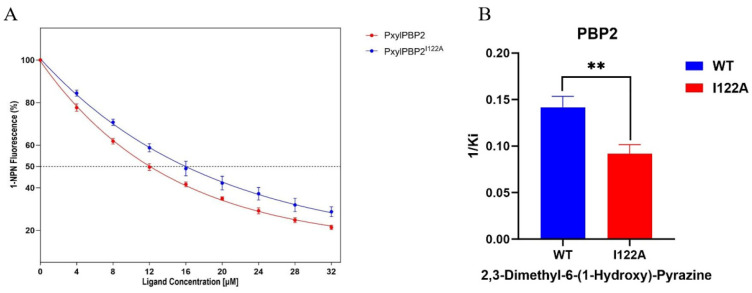
Binding between PxylPBP2 and 2,3-dimethyl-6-(1-hydroxy)-pyrazine. (**A**) Ligand binding curves of PxylPBP2 and PxylPBP2^I122A^. (**B**) Significance analysis for the difference between the Ki values of the ligand binding curves of PxylPBP2 and PxylPBP2^I122A^. Differences between groups are denoted by **, with *p* < 0.01 representing the threshold for statistical significance.

Subsequently, the PxylPBP2^I122A^ mutant was obtained with a mass weight between 15 and 25 kDa, as validated and purified via a standard method (Figure S6). The Kd value between PxylPBP2^I122A^ and fluorescence 1-NPN was 3.01 μmol/L (Figure S7), showing a significant decrease in binding affinity compared with the wild PxylPBP2 (1.20 μmol/L). As shown in Figure 4A, the Ki value between 2,3-dimethyl-6-(1-hydroxy)-pyrazine and PxylPBP2^I122A^ was 10.71 μM, while the value between PxylPBP2 and the ligand was 7.13 μmol/L. There was a significant difference between mutant and wild PxylPBP2 (*p* < 0.01), confirming that the mutant of the I122 site had an obvious influence on the binding ability between 2,3-dimethyl-6-(1-hydroxy)-pyrazine and PxylPBP2 (Figure 4B).

### 3.5. Recognition Characteristics of PxylPBP2 for Some Common Volatile Compounds

**Figure 5:**
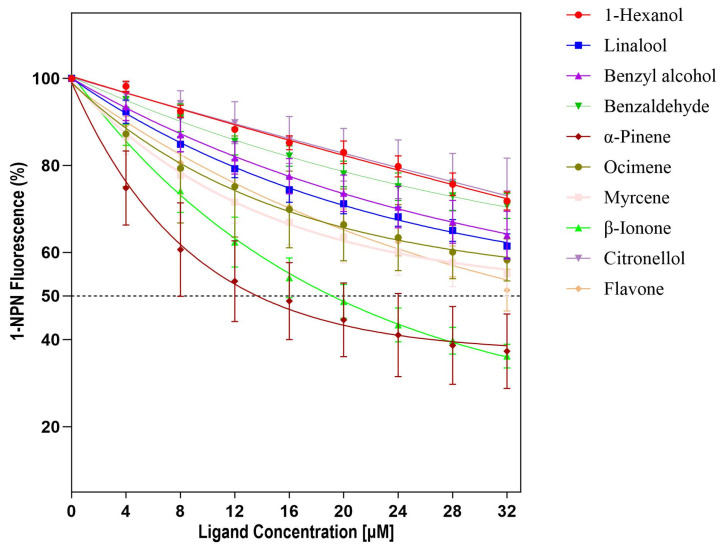
Ligand binding curves between PxylPBP2 and ten volatile compounds.

To gain an in-depth understanding of the recognition properties of PxylPBP2, the binding affinity between PxylPBP2 and ten common volatile compounds was assayed using fluorescence competitive binding. As shown in Figure 5, strong binding ability was found between PxylPBP2 and *α*-pinene and *β*-ionone, with Ki values of 10.54 μmol/L and 11.74 μmol/L, respectively. In addition, the Ki values were 28.30 μmol/L, 31.43 μmol/L, 35.72 μmol/L, and 49.27 μmol/L for binding affinity between PxylPBP2 and flavone, myrcene, ocimene, and linalool, respectively, which showed moderate binding ability. However, the Ki values for binding affinity between PxylPBP2 and 1-hexanol, benzyl alcohol, benzaldehyde, and *β*-citronellol were all higher than 50 μmol/L, suggesting no obvious binding occurred between PxylPBP2 and these compounds. These results provide an important basis for further studying the function of PxylPBP2 and its role in the recognition of volatile compounds.

## 4. Discussion

Due to the longstanding over-reliance on chemical pesticides for controlling the diamondback moth resistance to many insecticides, together with residue and environmental pollution problems, the situation has become ever more serious, posing a critical threat to agriculture and ecology [41]. The development and utilization of novel active ingredients has become a creative strategy for solving these problems, and these ingredients can also be used to achieve green control of the DBM. In our previous studies, 2,3-dimethyl-6-(1-hydroxy)-pyrazine showed obvious potential to act as a repellent for the DBM, with PxylPBP2 identified as the candidate molecular target, but the recognition mechanisms for pyrazines and other common volatile components have rarely been reported. OBPs are important organs used by insects to recognize xenobiotics, with *PxylPBP1* only being expressed in the *P. xylostella* antennae; this finding also determined that a change in expression level is closely related to mating status [42]. Simultaneously, the expression levels of OBPs in *Dendrolimus punctatus* are closely correlated with mating behavior [43]. Moreover, the NPC2 family proteins present in *Tetranychus urticae* showed an obvious relationship with omnivorous habits, suggesting that these proteins may play an important role in binding and transporting odor molecules from various biological sources, serving as potential carriers of chemical substances [44]. PstrOBP9 is a key protein for identifying isothiocyanates, as it is highly expressed in *Phyllotreta striolata* larvae and adults, indicating that a similar recognition mechanism was observed at different stages [45].

RNAi is a gene silencing mechanism triggered by *dsRNA* or *siRNA* that can lead to the loss of target gene function. By specifically knocking down the expression of candidate genes, the impact on key insect behaviors can be observed, which is an effective method for verifying the function of OBP genes. In this study, when the silencing rate of *PxylPBP2* was decreased 45.65%, the repellent activity of 2,3-dimethyl-6-(1-hydroxy)-pyrazine against the DBM was significantly reduced, confirming that PxylPBP2 was the insecticidal target. To evaluate the influence of *SlGOBP2* on resistance to chlorpyrifos in *Spodoptera litura*, *dsRNA* was injected into the larvae, significantly inhibiting the expression level of this gene, and the mortality rate increased after the RNAi procedure was completed [46]. Food consumption by the *Locusta migratoria* significantly decreased after *LmigOBP1* was silenced, and the EAG response to (Z)-3-hexenol, linalool, nonanal, decanal, and (Z)-3-hexenyl acetate was notably reduced [47]. When the expression level of *Obp83g-2* in *Bactrocera dorsalis* was downregulated using RNAi, the average EAG responses and oviposition behavior sensitivity to *γ*-octalactone were significantly decreased [48]. After 48 h, *OcomOBP11* dsRNA was injected using RNAi; responses of *Ophraella communa* Lesage adults to *β*-caryophyllene, *α*-pinene, camphene, octanal, and nonanal were significantly down-regulated, and the behavioral response of leaf beetles to camphene shifted from attraction to avoidance [49]. Furthermore, it was all known that specific OBPs function in diverse physiological processes beyond canonical olfactory pathways, behenic acid chemoreception determines dismemberment behavior via *SiApoLp-III*/*SiOBP15* in *Solenopsis invicta*, whereas *SiOBP15* or *SiOBP15*/*SiFABP5* recognition of linoleic acid inhibits dismemberment behaviour [50]. *OBP16* and *OBP18* in honey bees were found to be highly expressed in the antennae of bees engaging in high levels of hygiene behavior, including corpse removal [51]. In *Locusta migratoria*, *LmigCSP60* was found responsible for detecting and mediating avoidance behaviour to volatiles in fungal-contaminated food, while *LmigOBP11* was also found to respond to fungal volatiles [52,53].

The fluorescence competition binding assay is an important approach for studying the interaction between compounds and potential target proteins, as PxylPBP2 showed good binding affinity with 2,3-dimethyl-6-(1-hydroxy)-pyrazine using this method [38]. Nerolidol, *β*-ionone, trans-farnesol, and juvenile hormone III exhibit strong binding affinity with AlinOBP14 in *Adelphocoris lineolatus*, which can be used as a carrier not only for terpene compounds but also potentially for endogenous compounds [54]. The function of a protein reportedly largely depends on its spatial structure, so the microscale thermophoresis test and molecular dynamics simulation are important methods for clarifying the characteristics of insect proteins and interactions between the ligands [55]. *TcinOBP1* in *Tetranychus cinnabarinus* was significantly upregulated at 15 min and gradually decreased at 45 min post-ethyl oleate-treatment, and the MST test showed that the recombinant TcinOBP1 protein displayed a strong binding affinity to ethyl oleate (Kd = 32.3 μmol/L) [56]. Five insecticyanin genes (SeIns1-5) in *S. exigua* were identified, and molecular docking and microscale thermophoresis demonstrated that SeIns1 specifically binds to 2-cyhalothrin (Kd = 9.04 μmol/L) and chlorpyrifos (Kd = 105.24 μmol/L) with high affinity, suggesting its potential role in insecticide transport [57]. Simultaneously, SeApoD3/4 in *S. exigua* showed a high affinity for lambda-cyhalothrin and chlorpyrifos, and as identified via the MST test, these proteins play a possible role in resistance [58]. Moreover, *Tribolium castaneum* GSTe2 (TcGSTe2) has been implicated in metamorphosis, while molecular dynamics simulation confirmed the superior binding affinity and stability of the ZINC000111483109-TcGSTe2 complex, as supported by RMSD, RMSF (Root mean square fluctuation), the hydrogen bond number, and the total potential energy [59]. The ecdysone receptor (BtEcR) of *Bemisia tabaci* has previously been targeted to prevent fundamental developmental processes, with compounds ZINC08952607 and ZINC04264850 showing binding free energies of −170.156 kJ/mol and −200.349 kJ/mol, respectively, in complex with BtEcR, as determined using molecular dynamics simulation [60]. Moreover, *Anopheles gambiae* GST (Aggst1-2) underwent small conformational changes after ligand docking to the protein, facilitating the catalytic reaction using molecular dynamics simulation [61].

In addition, amino acid site-directed mutagenesis technology can modify the residues of target proteins, as widely used in our analysis of the function and binding characteristics, as certain key amino acids have a significant impact on their biological activity. When the lysine residue of the C-terminal in LYS123 (Lysine 123) of HarmOBP7 was replaced by methionine, the binding affinity of the mutant *Helicoverpa armigera* to the pheromone components Z-11-hexadecenal and Z-9-hexadecenal and their analogs was significantly decreased [62]. Three sites of HoblOBP1 in *Holotrichia oblita* can be used to create new mutants, as TYR111 can form hydrogen bonds with ASP107 (Aspartic acid 107) and HIS100 (Histidine 100), as used to fix hexyl benzoate. The distance between ILE800 and the ligand was shortest, which mainly affected the Van der Waals force and hydrophobic interaction, while MET48 was located at the bottom of the binding pocket, which influences the length of the ligand [63]. Three hydrogen-bonded residues were selected to obtain the mutants, which were determined based on the binding conformation of the protein and ligand according to the binding energy and potential hydrogen bonds via a molecular docking model [64]. Hence, in our study, we systematically analyzed the potential binding sites between PxylPBP2 and 2,3-dimethyl-6-(1-hydroxy)-pyrazine based on the molecular docking simulation results; isoleucine in No. 122 was confirmed to play an important role in the response of DBM to the repellent activity of pyrazine by comparing the change in binding energy after isoleucine was replaced with alanine.

## 5. Conclusions

The results of this study demonstrate that PxylPBP2 is the molecular target for the repellent activity of 2,3-dimethyl-6-(1-hydroxy)-pyrazine against *P. xylostella*, and ILE122 was an important target site, as validated via comparison with the mutant via the fluorescence competitive binding experiment. Simultaneously, the binding mechanism and characteristics of PxylPBP2 and common volatile components were clarified, such as *α*-pinene and *β*-ionone. Future studies should focus on developing novel active ingredients using the PxylPBP2 as a target, which will provide a creative strategy for delaying the development of resistance and achieving green control of *P. xylostella*.

## Data Availability

The data that support the findings of this study are available in the Supplementary Material of this article.

## Supplementary Materials

The following supporting information can be downloaded at https://www.mdpi.com/article/10.3390/insects17070708/s1, Figure S1: Silencing efficiency of the PxylPBP2 in the *P. xylostella*; Figure S2: Expression and purification of 1104YS-1-His (A), 104YS-2-His (B) protein and related original electrophoresis images (C, D). First channel was the marker, the second was the total protein of induced bacteria without IPTG, the third was the total protein of induced bacteria with IPTG, the forth was the total protein of supernate with IPTG, and the fifth was purification protein; Figure S3: Hydrogen interaction between 2,3-dimethyl-6-(1-hydroxy)-pyrazine and olfactory system protein; Figure S4: Van der Waals interaction between 2,3-dimethyl-6-(1-hydroxy)-pyrazine and olfactory system protein; Figure S5: Electrostatic interaction between 2,3-dimethyl-6-(1-hydroxy)-pyrazine and olfactory system protein; Figure S6: SDS-PAGE detection of PxylPBP2^I122A^ (A) and related original electrophoresis image (B). M, Protein marker; 1, Induction of expression product without IPTG; 2, Induction of expression product with IPTG; 3, Supernatant of expression product; 4, Precipitation of expression product; 5, Purification of mutant protein; Figure S7: Binding affinity of PxylPBP2^I122A^ and 1-NPN fluorescent probe; Table S1: Primers used for synthesis to dsRNA; Table S2: PCR amplification reaction system; Table S3: Reaction system for the synthesis to dsRNA; Table S4: PCR amplification reaction system of PxylPBP2^I122A^ site-directed mutation; Table S5: Digestion reaction system; Table S6: Recombination reaction system; Table S7: Volatile compounds used to measure the recognition characteristic of PxylPBP2; Table S8: Binding energy between PxylPBP2 and seven mutation sites of ligand.

## References

[1] You M., Ke F., You S., Wu Z., Liu Q., He W., Baxter S.W., Yuchi Z., Vasseur L., Gurr G.M. (2020). Variation among 532 genomes unveils the origin and evolutionary history of a global insect herbivore. Nat. Commun..

[2] Ma C.S., Zhang W., Peng Y., Zhao F., Chang X.Q., Xing K., Zhu L., Ma G., Yang H.P., Rudolf V.H.W. (2021). Climate warming promotes pesticide resistance through expanding overwintering range of a global pest. Nat. Commun..

[3] Shakeel M., Farooq M., Nasim W., Akram W., Khan F.Z.A., Jaleel W., Zhu X., Yin H., Li S., Fahad S. (2017). Environment polluting conventional chemical control compared to an environmentally friendly IPM approach for control of diamondback moth, *Plutella xylostella* (L.), in China, a review. Environ. Sci. Pollut. Res..

[4] Wang X., Wang R., Yang Y., Wu S., O’Reilly A.O., Wu Y. (2016). A point mutation in the glutamate-gated chloride channel of *Plutella xylostella* is associated with resistance to abamectin. Insect Mol. Biol..

[5] Wang X., Li X., Shen A., Wu Y. (2016). Baseline susceptibility of the diamondback moth (Lepidoptera, Plutellidae) to chlorantraniliprole in China. J. Econ. Entomol..

[6] Li X., Liu Z., Lv X., Liu X., Li Y., Tian Z., Zhang Y., Liu J. (2025). Molecular mechanism of λ-cyhalothrin detoxification by a delta-class glutathione *S*-transferase (PxGSTD3) from *Plutella xylostella*. J. Agric. Food Chem..

[7] Oplopoiou M., Elias J., Slater R., Bass C., Zimmer C.T. (2024). Characterization of emamectin benzoate resistance in the diamondback moth, *Plutella xylostella* (Lepidoptera, Plutellidae). Pest Manag. Sci..

[8] Nitta A., Boopathi T., Ankireddy J.R. (2025). Evaluation of insecticide resistance and associated enzyme activity in field populations of Diamondback Moth (*Plutella xylostella*). J. Appl. Toxicol..

[9] Li X., Li R., Zhu B., Gao X., Liang P. (2018). Overexpression of cytochrome P450 CYP6BG1 may contribute to chlorantraniliprole resistance in *Plutella xylostella* (L.). Pest Manag. Sci..

[10] Wu Q.J., Zhang S.F., Yao J.L., Xu B.Y., Wang S.L., Zhang Y.J. (2012). Management of diamondback moth, *Plutella xylostella* (Lepidoptera, Plutellidae) by mating disruption. Insect Sci..

[11] Shanmugapriya V., Edward Y.S.J.T., Kannan M., Kumar S.M., Ramanathan A. (2025). Inheritance of resistance and fitness cost parameters to cyantraniliprole in the resistant and susceptible strains of *Plutella xylostella* L.. Sci. Rep..

[12] Kolani L., Sanda K., Agboka K., Mawussi G., Koba K., Djouaka R. (2016). Investigation of insecticidal activity of blend of essential oil of *Cymbopogon schoenanthus* and neem oil on *Plutella xylostella* (Lepidoptera, Plutellidae). J. Essent. Oil Bear. Plants.

[13] Tavares W.R., Barreto M.D.C., Seca A.M. (2021). Aqueous and ethanolic plant extracts as bio-insecticides—Establishing a bridge between raw scientific data and practical reality. Plants.

[14] Li J., Zhou R., Han Q., Hui T., Qu Y., Yu S., Liang P., Ma Z., Gao Y. (2026). Study of natural turpentine-derived molecules as potential GOBP target indicators to control *Plutella xylostella*. J. Agric. Food Chem..

[15] Wang S., Li S.C., Cheng F.S., Ren T., Mei D.H., Gao K., Song Q.Y. (2022). Antifungal, repellency, and insecticidal activities of *Cymbopogon distans* and *Ruta graveolens* essential oils and their main chemical constituents. Chem. Biodivers..

[16] Song C., Zhao J., Zheng R., Hao C., Yan X. (2022). Chemical composition and bioactivities of thirteen non-host plant essential oils against *Plutella xylostella* L. (Lepidoptera, Plutellidae). J. Asia-Pac. Entomol..

[17] Peres L.L., Sobreiro A.I., Couto I.F., Silva R.M., Pereira F.F., Heredia-Vieira S.C., Cardoso C.A., Mauad M., Scalon S.P., Verza S.S. (2017). Chemical compounds and bioactivity of aqueous extracts of Alibertia spp. in the control of *Plutella xylostella* L. (Lepidoptera, Plutellidae). Insects.

[18] Ferreira E.A., de Souza S.A., Domingues A., Da Silva M.M.M., Padial I.M.P.M., de Carvalho E.M., Cardoso C.A.L., da Silva S.V., Mussury R.M. (2020). Phytochemical screening and bioactivity of Ludwigia spp. in the control of *Plutella xylostella* (Lepidoptera, Plutellidae). Insects.

[19] Yotavong P., Boonsoong B., Pluempanupat W., Koul O., Bullangpoti V. (2015). Effects of the botanical insecticide thymol on biology of a braconid, *Cotesia plutellae* (Kurdjumov), parasitizing the diamondback moth, *Plutella xylostella* L.. Int. J. Pest Manag..

[20] Peng J., Chen Z., Chen X., Zheng R., Lu S., Seyab M., Yang F., Li Q., Tang Q. (2023). Insecticidal potential of a *Consolida ajacis* extract and its major compound (ethyl linoleate) against the diamondback moth, *Plutella xylostella*. Pestic. Biochem. Physiol..

[21] Christiaens O., Sweet J., Dzhambazova T., Urru I., Smagghe G., Kostov K., Arpaia S. (2022). Implementation of RNAi-based arthropod pest control: Environmental risks, potential for resistance and regulatory considerations. J. Pest Sci..

[22] Bettencourt R., Terenius O., Faye I. (2002). *Hemolin* gene silencing by ds-RNA injected into *Cecropia* pupae is lethal to next generation embryos. Insect Mol. Biol..

[23] Li Z., Liu Y., Liang Y., Pan T., Liu J. (2026). RNA interference-based pesticides: Mechanism, application, and commercialization in sustainable pest management. Pestic. Biochem. Physiol..

[24] Fleischer J., Pregitzer P., Breer H., Krieger J. (2018). Access to the odor world, olfactory receptors and their role for signal transduction in insects. Cell. Mol. Life Sci..

[25] Del Mármol J., Yedlin M.A., Ruta V. (2021). The structural basis of odorant recognition in insect olfactory receptors. Nature.

[26] Yuan T., Wang H., Zhang Q.H., Wickham J.D., Zhang Y.N., Gu T., Zhang L. (2025). General odorant binding protein 2 and odorant binding protein 36 facilitate the recognition of adult sex pheromone components by *Hyphantria cunea* larvae. Insect Sci..

[27] Haverkamp A., Hansson B.S., Knaden M. (2018). Combinatorial codes and labeled lines, how insects use olfactory cues to find and judge food, mates, and oviposition sites in complex environments. Front. Physiol..

[28] Robertson H.M. (2019). Molecular evolution of the major arthropod chemoreceptor gene families. Annu. Rev. Entomol..

[29] Hekmat-Scafe D.S., Scafe C.R., McKinney A.J., Tanouye M.A. (2002). Genome-wide analysis of the odorant-binding protein gene family in *Drosophila melanogaster*. Genome Res..

[30] Vieira F.G., Rozas J. (2011). Comparative genomics of the odorant-binding and chemosensory protein gene families across the Arthropoda, origin and evolutionary history of the chemosensory system. Genome Biol. Evol..

[31] Zhang Z.C., Wang M.Q., Lu Y.B., Zhang G. (2009). Molecular characterization and expression pattern of two general odorant binding proteins from the diamondback moth, *Plutella xylostella*. J. Chem. Ecol..

[32] Sun M., Liu Y., Wang G. (2013). Expression patterns and binding properties of three pheromone binding proteins in the diamondback moth, *Plutella xyllotella*. J. Insect Physiol..

[33] Zhu J., Pelosi P., Liu Y. (2016). Ligand-binding properties of three odorant-binding proteins of the diamondback moth *Plutella xylostella*. J. Integr. Agric..

[34] Cai L.J., Zheng L.S., Huang Y.P., Xu W., You M.S. (2021). Identification and characterization of odorant binding proteins in the diamondback moth, *Plutella xylostella*. Insect Sci..

[35] Wang P., Liu M., Lv C., Tian Z., Li R., Li Y., Zhang Y., Liu J. (2024). Identifying the key role of *Plutella xylostella* general odorant binding protein 2 in perceiving a larval attractant, (E, E)-2, 6-farnesol. J. Agric. Food Chem..

[36] Zhang Y., Wang B., Zhou Y., Liao M., Sheng C., Cao H., Gao Q. (2023). Identification and characterization of odorant receptors in *Plutella xylostella* antenna response to 2,3-dimethyl-6-(1-hydroxy)-pyrazine. Pestic. Biochem. Physiol..

[37] Wang B., Zhang Y., Wei Y., Liao M., Cao H., Gao Q. (2024). Functional analysis of three odorant receptors in *Plutella xylostella* response to repellent activity of 2,3-dimethyl-6-(1-hydroxy)-pyrazine. Pestic. Biochem. Physiol..

[38] Wang B., Zhang Y., Zhang C., Liao M., Cao H., Gao Q. (2024). Identification and functional characterization of two antenna-specifc odorant-binding proteins in *Plutella xylostella* response to 2,3-dimethyl-6-(1-hydroxy)-pyrazine. Int. J. Biol. Macromol..

[39] Stein J.A., Ianeselli A., Braun D. (2021). Kinetic microscale thermophoresis for simultaneous measurement of binding affinity and kinetics. Angew. Chem..

[40] Hreusova M., Novakova O., Brabec V. (2020). Thermodynamic insights by microscale thermophoresis into translesion DNA synthesis catalyzed by DNA polymerases across a lesion of antitumor platinum–acridine complex. Int. J. Mol. Sci..

[41] Xu J., Wang Z., Wang Y., Ma H., Zhu H., Liu J., Zhou Y., Deng X., Zhou X. (2020). ABCC2 participates in the resistance of *Plutella xylostella* to chemical insecticides. Pestic. Biochem. Physiol..

[42] Zhang Z.C., Wang M.Q., Zhang G. (2009). Molecular cloning and expression of pheromone-binding protein1 from the diamondback moth, *Plutella xylostella*. Entomol. Exp. ET Appl..

[43] Zhang S.F., Zhang Z., Kong X.B., Wang H.B., Liu F. (2018). Dynamic changes in chemosensory gene expression during the *Dendrolimus punctatus* mating process. Front. Physiol..

[44] Zhu J., Renzone G., Arena S., Dani F.R., Paulsen H., Knoll W., Cambillau C., Scaloni A., Pelosi P. (2021). The odorant-binding proteins of the spider mite *Tetranychus urticae*. Int. J. Mol. Sci..

[45] Xiao Y., Sun L., Wu Y., Wang Q., Zhang Y., Jing X., Li Z. (2024). The larvae of *Phyllotreta striolata* share the same olfactory cues for locating Brassicaceae plant with conspecific adults. J. Pest Sci..

[46] Sun Z., Wang R., Du Y., Gao B., Gui F., Lu K. (2021). Olfactory perception of herbicide butachlor by GOBP2 elicits ecdysone biosynthesis and detoxification enzyme responsible for chlorpyrifos tolerance in *Spodoptera litura*. Environ. Pollut..

[47] Li J., Zhang L., Wang X. (2016). An Odorant-binding protein involved in perception of host plant odorants in locust *Locusta migratoria*. Arch. Insect Biochem. Physiol..

[48] Chen X., Lei Q., Liang C., Wang J., Jiang H. (2025). A case study on the γ-octalactone induced expression of *Obp83g-2* in *Bactrocera dorsalis* (Hendel) revealed the transcriptional regulation of insect odorant binding protein. Commun. Biol..

[49] Yue Y., Ma C., Zhang Y., Ma W.H., Wang J.J., Tian Z.Y., Chen G.M., Li R.M., Li J.H., Yang J.F. (2025). Functional analysis of *Ophraella communa* Lesage *OcomOBP11* in recognition of *Ambrosia artemisiifolia* L. volatiles. Pestic. Biochem. Physiol..

[50] Zhang W., Chen X., Tian J., Schal C., Mohamed A., Zang L.S., Xia Y., Keyhani N.O. (2025). An odorant-binding protein functions in fire ant social immunity interfacing with innate immunity. Open Biol..

[51] Guarna M.M., Melathopoulos A.P., Huxter E., Iovinella I., Parker R., Stoynov N., Tam A., Moon K.M., Chan Q.W.T., Pelosi P. (2014). A search for protein biomarkers links olfactory signal transduction to social immunity. Farm Animal Proteomics.

[52] Zheng R., Xie M., Keyhani N.O., Xia Y. (2023). An insect chemosensory protein facilitates locust avoidance to fungal pathogens via recognition of fungal volatiles. Int. J. Biol. Macromol..

[53] Zhang W., Xie M., Eleftherianos I., Mohamed A., Cao Y., Song B., Zang L.S., Jia C., Bian J., Keyhani N.O. (2023). An odorant binding protein is involved in counteracting detection-avoidance and Toll-pathway innate immunity. J. Adv. Res..

[54] Tian W., Zhang T., Gu S., Guo Y., Gao X., Zhang Y. (2021). OBP14 (Odorant-Binding Protein) sensing in *Adelphocoris lineolatus* based on peptide nucleic acid and graphene oxide. Insects.

[55] Arnold K., Bordoli L., Kopp J., Schwede T. (2006). The SWISS-MODEL workspace, a web-based environment for protein structure homology modelling. Bioinformatics.

[56] Chen Y.J., Zhang T.Y., Wan N.F., Zhao J., Jiang J.X., Ji X.Y. (2025). Role of odorant binding proteins in the response of *Tetranychus cinnabarinus* to repellent activity of ethyl oleate. Insect Biochem. Mol. Biol..

[57] Liu K., Jiang W., Guo H., Rao C., Su J. (2025). The identification of insecticyanin with high insecticide-binding affinity in *Spodoptera exigua*. Pestic. Biochem. Physiol..

[58] Liu K., Guo H., Jiang W., Rao C., Su J. (2025). Identification and structural analysis of apolipoprotein D with high insecticide-binding affinity in *Spodoptera exigua*. J. Agric. Food Chem..

[59] Kim K., Li C., Li B. (2025). Identification of novel inhibitor against *Tribolium castaneum* GSTe2, protein modelling, structure-based virtual screening and molecular dynamics simulation. Mol. Simul..

[60] Mangat H.K., Rani M., Pathak R.K., Yadav I.S., Utreja D., Chhuneja P.K., Chhuneja P. (2022). Virtual screening, molecular dynamics and binding energy-MM-PBSA studies of natural compounds to identify potential EcR inhibitors against *Bemisia tabaci* Gennadius. PLoS ONE.

[61] Wang Y., Zheng Q.C., Zhang J.L., Cui Y.L., Xue Q., Zhang H.X. (2013). Highlighting a π–π interaction, a protein modeling and molecular dynamics simulation study on *Anopheles gambiae* glutathione *S*-transferase 1-2. J. Mol. Model..

[62] Sun Y.L., Huang L.Q., Pelosi P., Wang C.Z. (2013). A lysine at the C-terminus of an odorant-binding protein is involved in binding aldehyde pheromone components in two Helicoverpa species. PLoS ONE.

[63] Zhuang X., Wang Q., Wang B., Zhong T., Cao Y., Li K., Yin J. (2014). Prediction of the key binding site of odorant-binding protein of *Holotrichia oblita* Faldermann (Coleoptera: Scarabaeida). Insect Mol. Biol..

[64] Yin N.N., Yang A.J., Wu C., Xiao H.Y., Guo Y.R., Liu N.Y. (2022). Genome-wide analysis of odorant-binding proteins in *Papilio xuthus* with focus on the perception of two PxutGOBPs to host odorants and insecticides. J. Agric. Food Chem..

