# Supplementary material for: Functional Analysis of PxylPBP2 Responding to Repellent Activity of Natural Pyrazine Against Diamondback Moth
Source: Insects. 2026 Jul 8;17(7):708. doi: 10.3390/insects17070708 (PMC13410483; doi:10.3390/insects17070708)
Supplement: Supplementary file 1 [file insects-17-00708-s001.zip › insects-4334587-supplementary.pdf]

Supplementary material

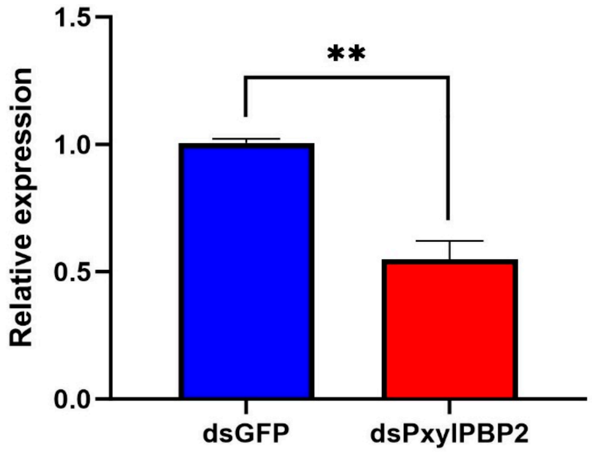

Figure S1 Silencing efficiency of the PxylPBP2 in the *P. xylostella*

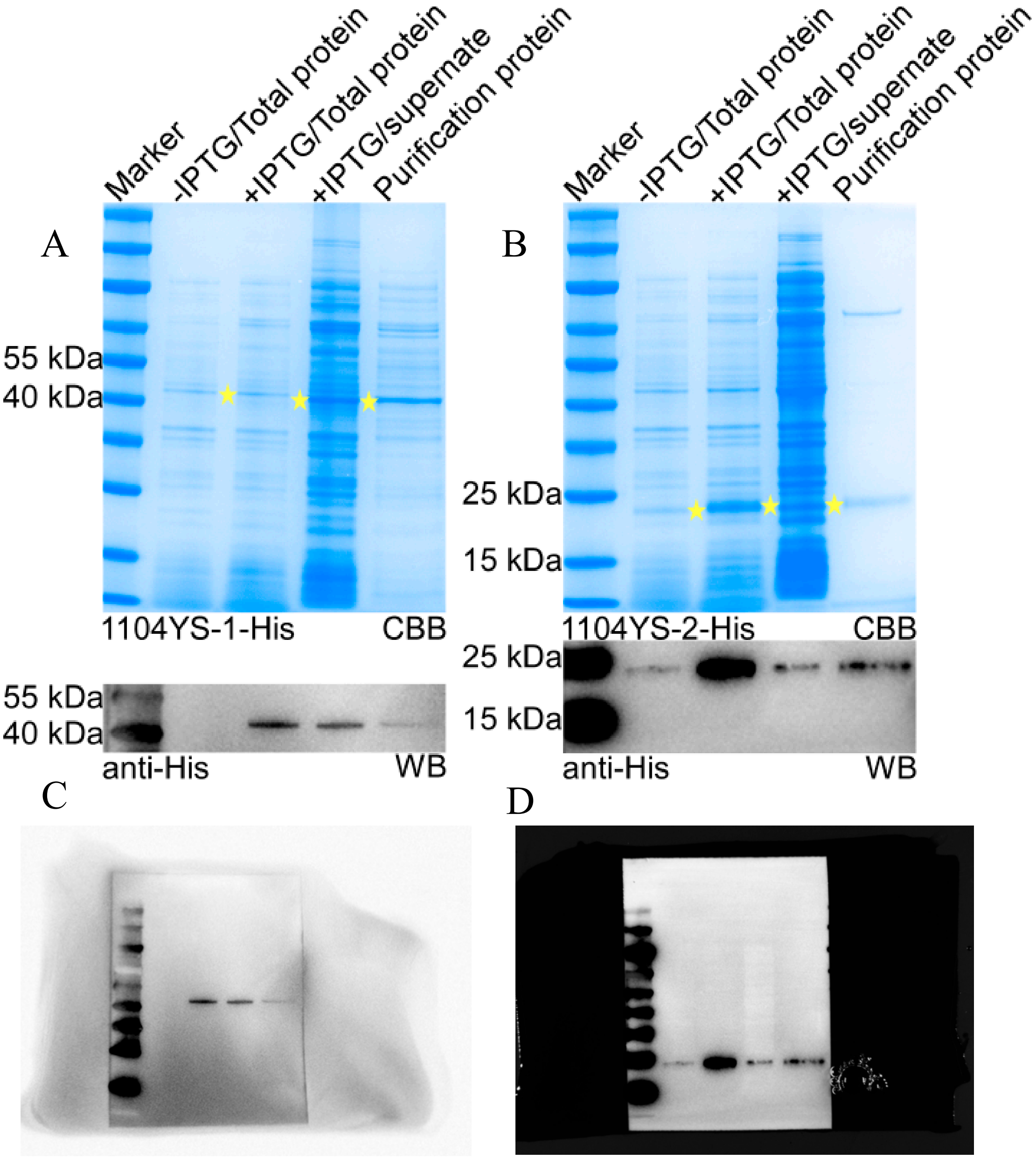

Figure S2 Expression and purification of 1104YS-1-His (A), 104YS-2-His (B) protein and related original electrophoresis images (C, D). First channel was the marker, the second was the total protein of induced bacteria without IPTG, the third was the total protein of induced bacteria with IPTG, the forth was the total protein of supernate with IPTG, and the fifth was purification protein

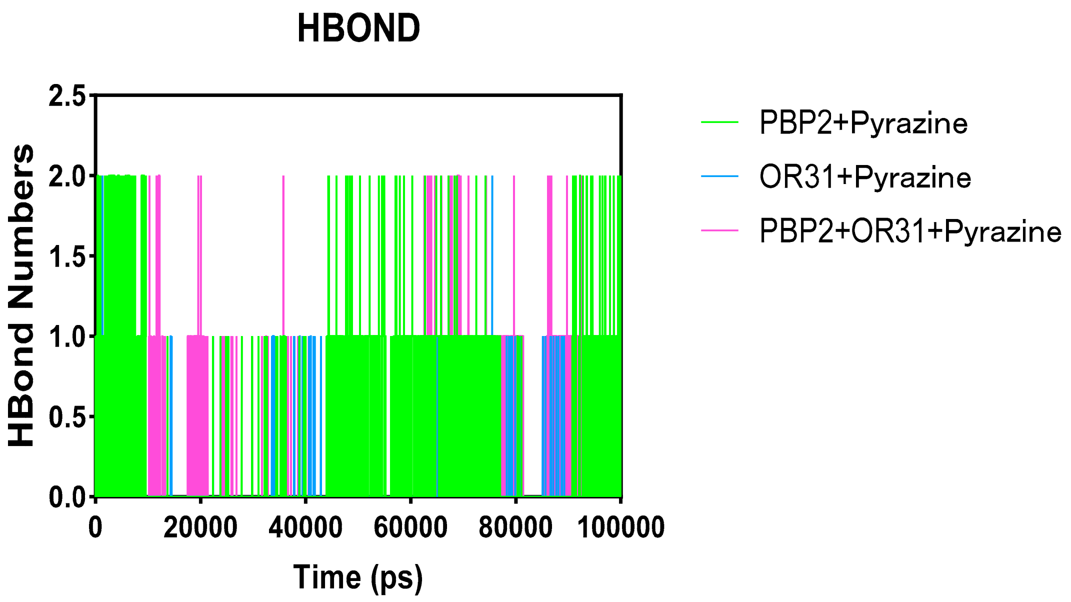

Figure S3 Hydrogen interaction between 2,3-dimethyl-6-(1-hydroxy)-pyrazine and olfactory system protein

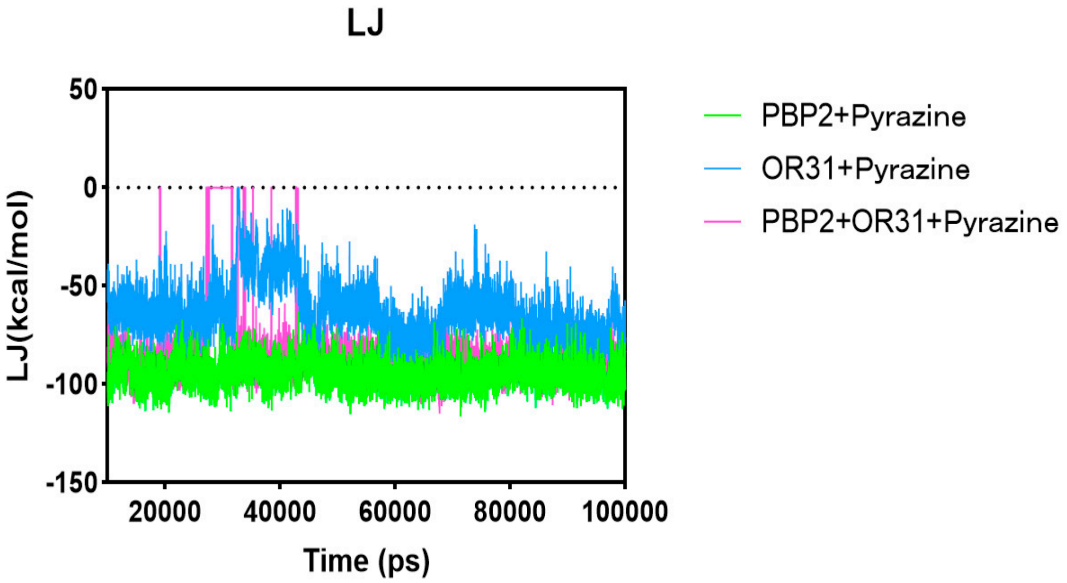

Figure S4 Van der Waals interaction between 2,3-dimethyl-6-(1-hydroxy)-pyrazine and olfactory system protein

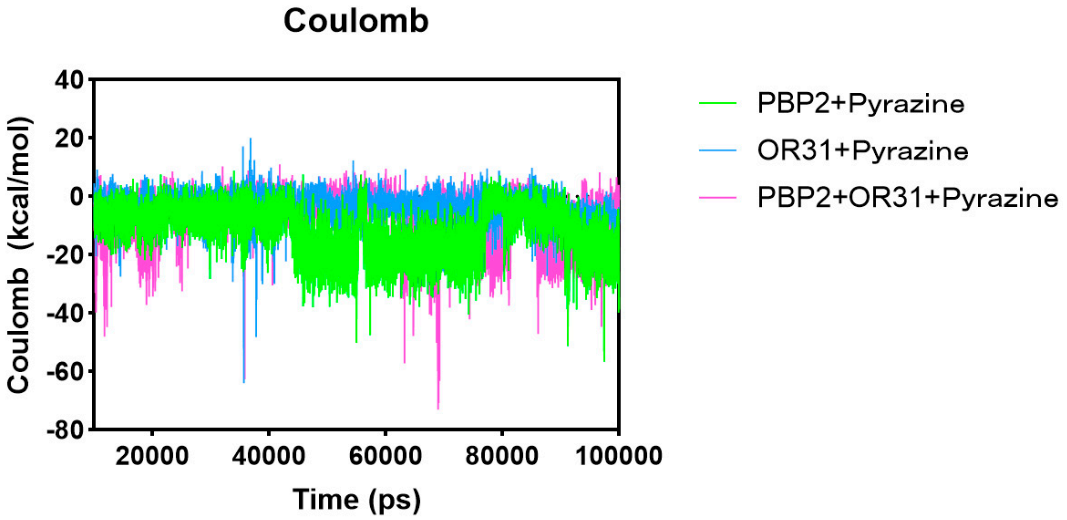

Figure S5 Electrostatic interaction between 2,3-dimethyl-6-(1-hydroxy)-pyrazine and olfactory system protein

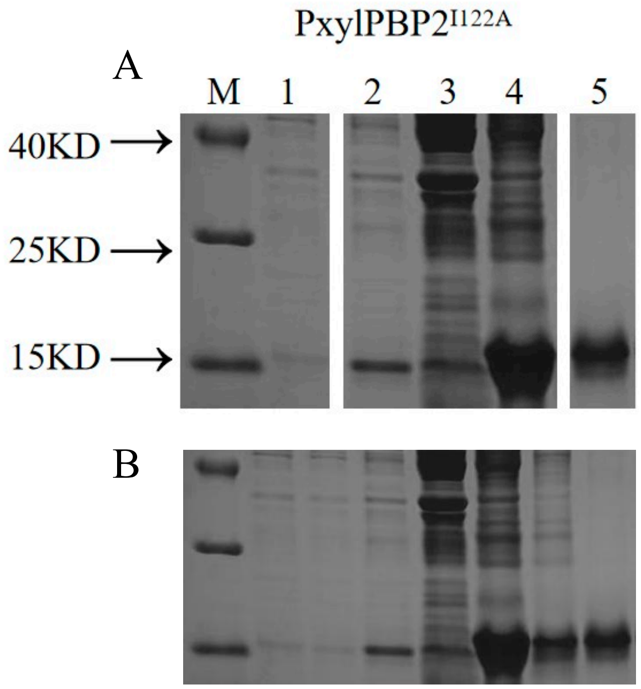

Figure S6 SDS-PAGE detection of PxylPBP2^{I122A} (A) and related original electrophoresis image (B). M, Protein marker; 1, Induction of expression product without IPTG; 2, Induction of expression product with IPTG; 3, Supernatant of expression product; 4, Precipitation of expression product; 5, Purification of mutant protein

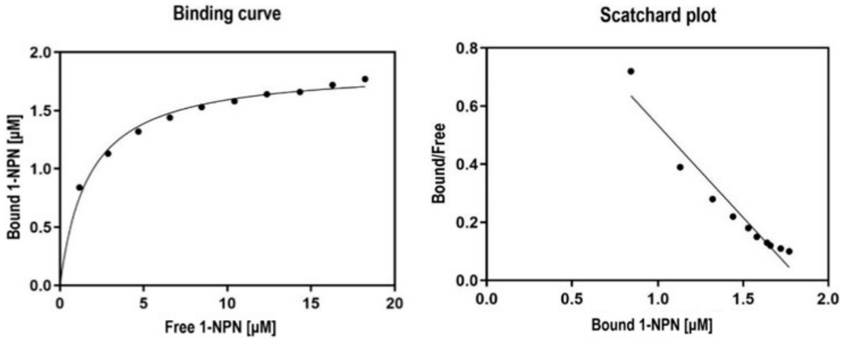

Figure S7 Binding affinity of PxylPBP2^{I122A} and 1-NPN fluorescent probe

Table S1. Primers used for synthesis to dsRNA

Gene	Sequences (Forward)	Sequences (Reverse)
dsPxylPBP2	TAATACGACTCACTATAGGG AGGACCTCAAGATGCACCAC	TAATACGACTCACTATAGGG TCAGTTCTTGAGCTCGGTGA
dsGFP	TAATACGACTCACTATAGGG AGTGCTTCAGCCGCTACCC	TAATACGACTCACTATAGGG GCGCTTCTCGTTGGGGTC

Note: the sequence annotated with underscore is T7 promoter.

Table S2. PCR amplification reaction system

Reagent	Volume
PrimeSTAR max premix	25 μL
Forward primer（10 μM）	2 μL
Reverse primer（10 μM）	2 μL
Template	1 μL
RNase free water	20 μL

Table S3. Reaction system for the synthesis to dsRNA

Reagent	Volume/Weight
RiboMAX^{TM} express T7 2 × buffer	10 μL
Enzyme mix, T7 express	2 μL
Product	1 μg
RNase free water	20 μL

Table S4. PCR amplification reaction system of PxylPBP2^{I122A} site-directed mutation

Reagent	Volume/Weight
2 × Max buffer	25 μL
dNTP mix (10 mM each)	1 μL
DNA template	1 ng
Forward primer	2 μL
Reverse primer	2 μL
Phanta max super-fidelity DNA polymerase	1 μL
RNase free water	To 50 μL

Table S5. Digestion reaction system

Reagent	Volume
Dpn I	1 μL
PCR amplification product	40 μL

Table S6. Recombination reaction system

Reagent	Volume or Weight
Dpn I digestion product	110 ng
5×CE II buffer	4 μL
Exnase II	2 μL
RNase free water	To 20 μL

Table S7. Volatile compounds used to measure the recognition characteristic of PxylPBP2

Substance	CAS No.	Relative molecular mass	Purity/%
1-Hexanol	111-27-3	102.17	99.00
Linalool	78-70-6	154.25	98.00
Benzyl alcohol	100-51-6	108.14	99.00
Benzaldehyde	100-52-7	106.12	99.00
α-Pinene	80-56-8	136.23	98.00
Ocimene	13877-91-3	136.23	90.00
Myrcene	123-35-3	136.23	90.00
β-Ionone	14901-07-6	192.3	97.00
β-Citronellol	106-22-9	156.27	95.00
Flavone	525-82-6	222.24	99.00

Table S8. Binding energy between PxylPBP2 and seven mutation sites of ligand

Ligand name	Binding energy (kcal/mol)	Ligand energy (kcal/mol)	Protein energy (kcal/mol)	Complex energy (kcal/mol)	Entropic energy (kcal/mol)
PxylPBP2-M33A	-19.21	37.95	-5606.21	-5587.47	17.47
PxylPBP2-L36A	-14.61	37.66	-5595.48	-5572.43	17.47
PxylPBP2-F40A	-21.29	39.03	-5610.44	-5592.69	17.48
PxylPBP2-W65A	-13.05	37.80	-5564.58	-5539.83	17.47
PxylPBP2-L80A	-11.90	37.33	-5601.32	-5575.88	17.47
PxylPBP2-I122A	-5.62	38.23	-5601.49	-5568.89	17.47
PxylPBP2-F146A	-15.69	38.37	-5594.43	-5571.76	17.47
